# Long Non-Coding RNA Profile in Genetic Symptomatic and Presymptomatic Frontotemporal Dementia: A GENFI Study

**DOI:** 10.3233/JAD-240557

**Published:** 2024-08-20

**Authors:** Maria Serpente, Chiara Fenoglio, Marina Arcaro, Tiziana Carandini, Luca Sacchi, Manuela Pintus, Emanuela Rotondo, Vittoria Borracci, Laura Ghezzi, Arabella Bouzigues, Lucy L. Russell, Phoebe H. Foster, Eve Ferry-Bolder, John C. van Swieten, Lize C. Jiskoot, Harro Seelaar, Raquel Sánchez Valle, Robert Laforce, Caroline Graff, Rik Vandenberghe, Alexandre de Mendonça, Pietro Tiraboschi, Isabel Santana, Alexander Gerhard, Johannes Levin, Sandro Sorbi, Markus Otto, Florence Pasquier, Simon Ducharme, Chris R. Butler, Isabelle Le Ber, Elizabeth Finger, Maria Carmela Tartaglia, Mario Masellis, James B. Rowe, Matthis Synofzik, Fermin Moreno, Barbara Borroni, Jonathan D. Rohrer, Andrea Arighi, Daniela Galimberti, Genetic FTD Initiative GENFI, Antonella Alberici, Sónia Afonso, Patricia Alves, Sarah Anderl-Straub, Anna Antonell, Mircea Balasa, Myriam Barandiaran, Nuria Bargalló, Robert Bartha, Benjamin Bender, Alexander Maximilian Bernhardt, Maxime Bertoux, Anne Bertrand, Valentina Bessi, Sandra Black, Giorgio Bocca, Martina Bocchetta, Sergi Borrego-Ecija, Alexis Brice, Rose Bruffaerts, Francesca R Buccellato, Emanuele Buratti, Valentina Cantoni, Paola Caroppo, David Cash, Miguel Castelo-Branco, Olivier Colliot, Rhian Convery, Thomas Cope, Tiago Costa-Coelho, Ioana Croitoru, Agnès Camuzat, Marianna D’Anca, Liset de Boer, Julie de Houwer, Vincent Deramecourt, João Durães, Giuseppe Di Fede, Camilla Ferrari, Graziana Florio, Marta Frascotti, Morris Freedman, Aurélie Funkiewiez, Alazne Gabilondo, Roberto Gasparotti, Giorgio Giaccone, Lucia Giannini, Sophie Goldsmith, Lisa Graf, Vesna Jelic, Ron Keren, Johanna Krüger, Gregory Kuchcinski, Tobias Langheinrich, Thibaud Lebouvier, Maria João Leitão, João Lemos, Marisa Lima, Albert Lladó, Gemma Lombardi, Jolina Lombardi, Maura Malpetti, Carolina Maruta, David Mengel, Gabriel Miltenberger, Sara Mitchell, Maxime Montembault, Benedetta Nacmias, Mattias Nilsson, Linn Öijerstedt, Jaume Olives, Janne M. Papma, Yolande Pijnenburg, Koen Poesen, Cristina Polito, Jackie Poos, Enrico Premi, Sara Prioni, Catharina Prix, Veronica Redaelli, Timothy Rittman, Rosa Rademakers, Daisy Rinaldi, Ekaterina Rogaeva, Adeline Rollin, Pedro Rosa-Neto, Maria Rosario Almeida, Giacomina Rossi, Kiran Samra, Dario Saracino, Sabrina Sayah, Elio Scarpini, Sonja Schönecker, Christen Shoesmith, Frederico Simões do Couto, Anna Stockbauer, Miguel Tábuas-Pereira, David Tang-Wai, Melissa Taheri Rydell, Mikel Tainta, David L Thomas, Mathieu Vandenbulcke, Philip Van Damme, Rick van Minkelen, Ana Verdelho, Henrik Viklund, Roberto Vimercati, Annick Vogels, Olivia Wagemann, Elisabeth Wlasich

**Affiliations:** Centre for Neurodegenerative Disorders, Department of Clinical and Experimental Sciences, University of Brescia, Brescia, Italy; Instituto Ciencias Nucleares Aplicadas a Saude, Universidade de Coimbra, Coimbra, Portugal; Instituto de Investigación Sanitaria Biogipuzkoa, Neurosciences Area, Group of Neurodegenerative Diseases, San Sebastian, Spain; Department of Neurology, University of Ulm, Ulm, Germany; Alzheimer’s disease and Other Cognitive Disorders Unit, Neurology Service, Hospital Clínic, Barcelona, Spain; Alzheimer’s disease and Other Cognitive Disorders Unit, Neurology Service, Hospital Clínic, Barcelona, Spain; Cognitive Disorders Unit, Department of Neurology, Donostia University Hospital, San Sebastian, Gipuzkoa, Spain; Imaging Diagnostic Center, Hospital Clínic, Barcelona, Spain; Department of Medical Biophysics, The University of Western Ontario, London, Ontario, Canada; Department of Diagnostic and Interventional Neuroradiology, University of Tübingen, Tübingen, Germany; Neurologische Klinik, Ludwig-Maximilians-Universität München, Munich, Germany; Inserm 1172, Lille, France; CHU, CNR-MAJ, Labex Distalz, LiCEND Lille, France; Sorbonne Université, Paris Brain Institute – Institut du Cerveau – ICM, Inserm U1127, CNRS UMR 7225, AP-HP - Hôpital Pitié-Salpêtrière, Paris, France; Department of Neuroscience, Psychology, Drug Research and Child Health, University of Florence, Florence, Italy; Sunnybrook Health Sciences Centre, Sunnybrook Research Institute, University of Toronto, Toronto, Canada; University of Milan, Fondazione IRCCS Ca’ Granda Ospedale Maggiore Policlinico, Neurodegenerative Diseases Unit, Milan, Italy; Department of Neurodegenerative Disease, Dementia Research Centre, UCL Queen Square Institute of Neurology, London, UK; Alzheimer’s disease and Other Cognitive Disorders Unit, Neurology Service, Hospital Clínic, Barcelona, Spain; Sorbonne Université, Paris Brain Institute – Institut du Cerveau – ICM, Inserm U1127, CNRS UMR 7225, AP-HP - Hôpital Pitié-Salpêtrière, Paris, France; Department of Biomedical Sciences, University of Antwerp, Antwerp, Belgium; Biomedical Research Institute, Hasselt University, 3500 Hasselt, Belgium; Dept. of Biomedical, Surgical and Dental Sciences, University of Milan, Milan, Italy; Fondazione Ca’ Granda, IRCCS Ospedale Maggiore Policlinico, Milan, Italy; ICGEB Trieste, Italy; Valentina Cantoni Centre for Neurodegenerative Disorders, Department of Clinical and Experimental Sciences, University of Brescia, Brescia, Italy; Centre for Neurodegenerative Disorders, Department of Clinical and Experimental Sciences, University of Brescia, Brescia, Italy; Fondazione IRCCS Istituto Neurologico Carlo Besta, Milano, Italy; Department of Neurodegenerative Disease, Dementia Research Centre, UCL Queen Square Institute of Neurology, London, UK; Faculty of Medicine, University of Coimbra, Coimbra, Portugal; Sorbonne Université, Paris Brain Institute – Institut du Cerveau – ICM, Inserm U1127, CNRS UMR 7225, AP-HP - Hôpital Pitié-Salpêtrière, Paris, France; Department of Neurodegenerative Disease, Dementia Research Centre, UCL Queen Square Institute of Neurology, London, UK; Cambridge University Hospitals NHS Trust, University of Cambridge, Cambridge, UK; Faculty of Medicine, University of Lisbon, Lisbon, Portugal; Instituto de Investigación Sanitaria Biogipuzkoa, Neurosciences Area, Group of Neurodegenerative Diseases, San Sebastian, Spain; Sorbonne Université, Paris Brain Institute – Institut du Cerveau – ICM, Inserm U1127, CNRS UMR 7225, AP-HP - Hôpital Pitié-Salpêtrière, Paris, France; Dept. of Biomedical, Surgical and Dental Sciences, University of Milan, Milan, Italy; Department of Neurology, Erasmus Medical Center, Rotterdam, Netherlands; Department of Neurology, Erasmus Medical Center, Rotterdam, Netherlands; Univ Lille, France; Inserm 1172, Lille, France; CHU, CNR-MAJ, Labex Distalz, LiCEND Lille, France; Neurology Department, Centro Hospitalar e Universitario de Coimbra, Coimbra, Portugal; Fondazione IRCCS Istituto Neurologico Carlo Besta, Milano, Italy; Department of Neuroscience, Psychology, Drug Research and Child Health, University of Florence, Florence, Italy; University of Milan, Milan, Italy; Fondazione IRCCS Ca’ Granda Ospedale Maggiore Policlinico, Neurodegenerative Diseases Unit, Milan, Italy; Baycrest Health Sciences, Rotman Research Institute, University of Toronto, Toronto, Canada; Sorbonne Université, Paris Brain Institute – Institut du Cerveau – ICM, Inserm U1127, CNRS UMR 7225, AP-HP - Hôpital Pitié-Salpêtrière, Paris, France; Daisy Sorbonne Université, Paris Brain Institute – Institut du Cerveau – ICM, Inserm U1127, CNRS UMR 7225, AP-HP - Hôpital Pitié-Salpêtrière, Paris, France; Cognitive Disorders Unit, Department of Neurology, Donostia University Hospital, San Sebastian, Gipuzkoa, Spain; Neuroradiology Unit, University of Brescia, Brescia, Italy; Fondazione IRCCS Istituto Neurologico Carlo Besta, Milano, Italy; Department of Neurology, Erasmus Medical Center, Rotterdam, Netherlands; Neuroimaging Analysis Centre, Department of Brain Repair and Rehabilitation, UCL Institute of Neurology, Queen Square, London, UK; Department of Neurodegenerative Diseases, Hertie-Institute for Clinical Brain Research and Center of Neurology, University of Tübingen, Tübingen, Germany; Division of Clinical Geriatrics, Karolinska Institutet, Stockholm; The University Health Network, Toronto Rehabilitation Institute, Toronto, Canada; Research Unit of Clinical Medicine, Neurology, University of Oulu, Oulu, Finland; Univ Lille, France; Inserm 1172, Lille, France; CHU, CNR-MAJ, Labex Distalz, LiCEND Lille, France; Division of Neuroscience and Experimental Psychology, Wolfson Molecular Imaging Centre, University of Manchester, Manchester, UK; Univ Lille, France; Inserm 1172, Lille, France; CHU, CNR-MAJ, Labex Distalz, LiCEND Lille, France; Centre of Neurosciences and Cell Biology, Universidade de Coimbra, Coimbra, Portugal; Faculty of Medicine, University of Coimbra, Coimbra, Portugal; Neurology Department, Centro Hospitalar e Universitario de Coimbra, Coimbra, Portugal; Alzheimer’s disease and Other Cognitive Disorders Unit, Neurology Service, Hospital Clínic, Barcelona, Spain; Department of Neuroscience, Psychology, Drug Research and Child Health, University of Florence, Florence, Italy; Department of Neurology, University of Ulm, Ulm, Germany; Department of Clinical Neurosciences, University of Cambridge, Cambridge, UK; Laboratory of Language Research, Centro de Estudos Egas Moniz, Faculty of Medicine, University of Lisbon, Lisbon, Portugal; Department of Neurodegenerative Diseases, Hertie-Institute for Clinical Brain Research and Center of Neurology, University of Tübingen, Tübingen, Germany; Faculty of Medicine, University of Lisbon, Lisbon, Portugal; Sunnybrook Health Sciences Centre, Sunnybrook Research Institute, University of Toronto, Toronto, Canada; Douglas Research Centre, Department of Psychiatry, McGill University, Montreal, Québec, Canada; Department of Neuroscience, Psychology, Drug Research and Child Health, University of Florence, Florence, Italy; Department of Clinical Neuroscience, Karolinska Institutet, Stockholm, Sweden; Department of Neurobiology, Care Sciences and Society; Center for Alzheimer Research, Division of Neurogeriatrics, Bioclinicum, Karolinska Institutet, Solna, Sweden; Alzheimer’s disease and Other Cognitive Disorders Unit, Neurology Service, Hospital Clínic, Barcelona, Spain; Department of Neurology, Erasmus Medical Center, Rotterdam, Netherlands; Amsterdam University Medical Centre, Amsterdam VUmc, Amsterdam, Netherlands; Laboratory for Molecular Neurobiomarker Research, KU Leuven, Leuven, Belgium; Department of Biomedical, Experimental and Clinical Sciences “Mario Serio”, Nuclear Medicine Unit, University of Florence, Florence, Italy; Department of Neurology, Erasmus Medical Center, Rotterdam, Netherlands; Stroke Unit, ASST Brescia Hospital, Brescia, Italy; Roberto Gasparotti Neuroradiology Unit, University of Brescia, Brescia, Italy; Fondazione IRCCS Istituto Neurologico Carlo Besta, Milano, Italy; Neurologische Klinik, Ludwig-Maximilians-Universität München, Munich, Germany; Fondazione IRCCS Istituto Neurologico Carlo Besta, Milano, Italy; Department of Clinical Neurosciences, University of Cambridge, Cambridge, UK; Center for Molecular Neurology, University of Antwerp, Belgium; Sorbonne Université, Paris Brain Institute – Institut du Cerveau – ICM, Inserm U1127, CNRS UMR 7225, AP-HP - Hôpital Pitié-Salpêtrière, Paris, France; Tanz Centre for Research in Neurodegenerative Diseases, University of Toronto, Toronto, Canada; CHU, CNR-MAJ, Labex Distalz, LiCEND Lille, France; Translational Neuroimaging Laboratory, McGill Centre for Studies in Aging, McGill University, Montreal, Québec, Canada; Faculty of Medicine, University of Coimbra, Coimbra, Portugal; Fondazione IRCCS Istituto Neurologico Carlo Besta, Milano, Italy; Department of Neurodegenerative Disease, Dementia Research Centre, UCL Queen Square Institute of Neurology, London, UK; Sorbonne Université, Paris Brain Institute – Institut du Cerveau – ICM, Inserm U1127, CNRS UMR 7225, AP-HP - Hôpital Pitié-Salpêtrière, Paris, France; Sorbonne Université, Paris Brain Institute – Institut du Cerveau – ICM, Inserm U1127, CNRS UMR 7225, AP-HP - Hôpital Pitié-Salpêtrière, Paris, France; Fondazione IRCCS Ca’ Granda Ospedale Maggiore Policlinico, Neurodegenerative Diseases Unit, Milan, Italy; Neurologische Klinik, Ludwig-Maximilians-Universität München, Munich, Germany; Department of Clinical Neurological Sciences, University of Western Ontario, London, Ontario, Canada; Faculdade de Medicina, Universidade Católica Portuguesa; Neurologische Klinik, Ludwig-Maximilians-Universität München, Munich, Germany; Neurology Department, Centro Hospitalar e Universitario de Coimbra, Coimbra, Portugal; Faculty of Medicine, University of Coimbra, Coimbra, Portugal; The University Health Network, Krembil Research Institute, Toronto, Canada; Department of Neurobiology, Care Sciences and Society; Center for Alzheimer Research, Division of Neurogeriatrics, Bioclinicum, Karolinska Institutet, Solna, Sweden; Instituto de Investigación Sanitaria Biogipuzkoa, Neurosciences Area, Group of Neurodegenerative Diseases, San Sebastian, Spain; Neuroimaging Analysis Centre, Department of Brain Repair and Rehabilitation, UCL Institute of Neurology, Queen Square, London, UK; Geriatric Psychiatry Service, University Hospitals Leuven, Belgium; Neuropsychiatry, Department of Neurosciences, KU Leuven, Leuven, Belgium; Neurology Service, University Hospitals Leuven, Belgium; Laboratory for Neurobiology, VIB-KU Leuven Centre for Brain Research, Leuven, Belgium; Department of Clinical Genetics, Erasmus Medical Center, Rotterdam, Netherlands; Department of Neurosciences and Mental Health, Centro Hospitalar Lisboa Norte - Hospital de Santa Maria & Faculty of Medicine, University of Lisbon, Lisbon, Portugal; Karolinska University Hospital Huddinge, Sweden; Fondazione IRCCS Ca’ Granda Ospedale Maggiore Policlinico, Neurodegenerative Diseases Unit, Milan, Italy; Department of Human Genetics, KU Leuven, Leuven, Belgium; Neurologische Klinik, Ludwig-Maximilians-Universität München, Munich, Germany; Neurologische Klinik, Ludwig-Maximilians-Universität München, Munich, Germany; a Fondazione Ca’ Granda, IRCCS Ospedale Maggiore Policlinico, Milan, Italy; b Department of Biomedical, Surgical and Dental Sciences, University of Milan, Milan, Italy; c Department of Neurodegenerative Disease, Dementia Research Centre, UCL Queen Square Institute of Neurology, London, UK; d Department of Neurology, Erasmus Medical Centre, Rotterdam, The Netherlands; e Alzheimer’s Disease and Other Cognitive Disorders Unit, Neurology Service, Hospital Clínic de Barcelona, Institut d’Investigacions Biomèdiques August Pi i Sunyer (IDIBAPS), Fundació Clínic per a la Recerca Biomèdica, Universitat de Barcelona, Barcelona, Spain; f Département des Sciences Neurologiques, Clinique Interdisciplinaire de Mémoire, CHU de Québec, and Faculté de Médecine, Université Laval, QC, Canada; g Department of Neurobiology, Care Sciences and Society; Center for Alzheimer Research, Division of Neurogeriatrics, Bioclinicum, Karolinska Institutet, Solna, Sweden; h Unit for Hereditary Dementias, Theme Inflammation and Aging, Karolinska University Hospital, Solna, Sweden; i Department of Neurosciences, Laboratory for Cognitive Neurology, KU Leuven, Leuven, Belgium; j Neurology Service, University Hospitals Leuven, Leuven, Belgium; k Leuven Brain Institute, KU Leuven, Leuven, Belgium; l Faculty of Medicine, University of Lisbon, Lisbon, Portugal; m Fondazione IRCCS Istituto Neurologico Carlo Besta, Milan, Italy; n University Hospital of Coimbra (HUC), Neurology Service, Faculty of Medicine, University of Coimbra, Coimbra, Portugal; o Center for Neuroscience and Cell Biology, Faculty of Medicine, University of Coimbra, Coimbra, Portugal; p Division of Psychology Communication and Human Neuroscience, Wolfson Molecular Imaging Centre, University of Manchester, Manchester, UK; q Department of Nuclear Medicine, Center for Translational Neuro- and Behavioral Sciences, University Medicine Essen, Essen, Germany; r Department of Geriatric Medicine, Klinikum Hochsauerland, Arnsberg, Germany; s Department of Neurology, Ludwig-Maximilians Universität München, Munich, Germany; t German Center for Neurodegenerative Diseases (DZNE), Munich, Germany; u Munich Cluster of Systems Neurology (SyNergy), Munich, Germany; v Department of Neurofarba, University of Florence, Italy; w IRCCS Fondazione Don Carlo Gnocchi, Florence, Italy; z Department of Neurology, University of Ulm, Germany; aa University of Lille, Lille, France; ab Inserm 1172, Lille, France; ac CHU, CNR-MAJ, Labex Distalz, LiCEND Lille, Lille, France; ad Department of Psychiatry, Douglas Mental Health University Institute, McGill University, Montreal, QC, Canada; ae McConnell Brain Imaging Centre, Montreal Neurological Institute, McGill University, Montreal, QC, Canada; af Nuffield Department of Clinical Neurosciences, Medical Sciences Division, University of Oxford, Oxford, UK; ag Department of Brain Sciences, Imperial College London, London, UK; ah Sorbonne Université, Paris Brain Institute – Institut du Cerveau – ICM, Inserm U1127, CNRS UMR 7225, AP-HP – Hôpital Pitié-Salpêtrière, Paris, France; ai Département de Neurologie, Centre de Référence Des Démences Rares Ou Précoces, IM2A, AP-HP – Hôpital Pitié-Salpêtrière, Paris, France; aj Département de Neurologie, AP-HP – Hôpital Pitié-Salpêtrière, Paris, France; ak Department of Clinical Neurological Sciences, University of Western Ontario, London, ON, Canada; al Tanz Centre for Research in Neurodegenerative Diseases, University of Toronto, Toronto, ON, Canada; am Sunnybrook Health Sciences Centre, Sunnybrook Research Institute, University of Toronto, Toronto, Canada; an Department of Clinical Neurosciences and Cambridge University Hospitals NHS Trust, University of Cambridge, Cambridge, UK; ao Department of Neurodegenerative Diseases, Hertie-Institute for Clinical Brain Research and Center of Neurology, University of Tübingen, Tübingen, Germany; ap German Center for Neurodegenerative Diseases (DZNE), Tubingen, Germany; aq Cognitive Disorders Unit, Department of Neurology, Donostia Universitary Hospital, San Sebastian, Spain; ar Biogipuzkoa Health Research Institute, Neurosciences Area, Group of Neurodegenerative Diseases, San Sebastian, Spain; as Center for Biomedical Research in Neurodegenerative Disease (CIBERNED), Carlos III Health Institute, Madrid, Spain; at Department of Clinical and Experimental Sciences, Neurology Unit, University of Brescia, Brescia, Italy

**Keywords:** Alzheimer’s disease, chromosome 9 open reading frame 72, frontotemporal dementia, long non-coding RNA, microtubule associated protein tau, progranulin

## Abstract

**Background:**

Long non-coding RNAs (lncRNAs) play crucial roles in gene regulation and are implicated in neurodegenerative diseases, including frontotemporal dementia (FTD). However, their expression patterns and potential as biomarkers in genetic FTD involving Chromosome 9 Open Reading Frame (*C9ORF72*), Microtubule Associated Protein Tau (*MAPT*), and Progranulin (*GRN*) genes are not well understood.

**Objective:**

This study aimed to profile the expression levels of lncRNAs in peripheral blood mononuclear cells collected within the GENetic Frontotemporal dementia Initiative (GENFI).

**Methods:**

Fifty-three lncRNAs were analyzed with the OpenArray Custom panel, in 131 patients with mutations in *C9ORF72*, *MAPT*, and *GRN*, including 68 symptomatic mutation carriers (SMC) and 63 presymptomatic mutation carriers (PMC), compared with 40 non-carrier controls (NC).

**Results:**

Thirty-eight lncRNAs were detectable; the relative expression of NEAT1 and NORAD was significantly higher in *C9ORF72* SMC as compared with NC. GAS5 expression was instead significantly lower in the *GRN* group versus NC. *MAPT* carriers showed no significant deregulations. No significant differences were observed in PMC. Disease duration did not correlate with lncRNA expression.

**Conclusions:**

NEAT1 and NORAD are upregulated in *C9ORF72* SMC and *GAS5* levels are downregulated in *GRN* SMC, underlining lncRNAs’ relevance in FTD and their potential for biomarker development. Further validation and mechanistic studies are crucial for clinical implications.

## INTRODUCTION

Long non-coding RNAs (lncRNAs) are RNA molecules encompassing more than 200 nucleotides. They do not possess canonical open reading frames^1^ and are not the product of alternative splicing of a coding gene.^2^ LncRNA regulates gene expression in neurodegenerative diseases. The majority of studies have been carried out in Alzheimer’s disease (AD). For example, it has been shown that the lncRNA amyloid-β cleaving enzyme-1 antisense, named *BACE1-AS*, is transcribed from the opposite strand of *BACE1* gene, and can form an RNA duplex. It binds to the BACE1 mRNA to improve its stability and translation, positively regulating the expression of BACE1 protein and promoting an increase in the cleavage of the amyloid-β protein precursor.^3^ Moreover, the lncRNA Brain Cytoplasmic 200 (BC200) was found to be upregulated in brain tissues from patients with AD and it has been shown that it enhances the production of amyloid protein through the regulation of *BACE1* expression.^4^ Few data on the role of lncRNAs in other neurodegenerative disorders, including frontotemporal dementia (FTD) are available, although it is known that they are involved in a number of common mechanisms, such as inflammation, oxidative damage and synaptic dysfunction (see^5^ for review).

FTD encompasses several clinical syndromes. The most common is the behavioral variant (bv)FTD, characterized by the development of behavioral disturbances, aggressiveness, lack of empathy, and decline in social conduct, followed by non-fluent variant primary progressive aphasia (nfvPPA) and semantic variant (sv)PPA. About 50% of FTD cases display a family history for dementia, often with dominant traits.^6^ At histopathology, all syndromes described are collectively classified as frontotemporal lobar degeneration (FTLD). According to the type of protein depositing, FTLD is classified into FTLD-Tau, FTLD-TAR DNA Binding protein (TDP)43, and FTLD fused in Sarcoma (FUS).^7^ Three major causal genes responsible for autosomal dominant inherited FTD have been discovered so far, including microtubule associated protein tau (*MAPT*), characterized by the deposition of tau protein in the brain, progranulin (*GRN*) and chromosome 9 open reading frame 72 (*C9ORF72*), both characterized by deposition of TDP-43. *MAPT* carriers quite often develop bvFTD with parkinsonism, whereas *GRN* mutations are associated with phenotypic heterogeneity, including the classical syndromes but also atypical presentations such as corticobasal syndrome (CBS) and progressive supranuclear palsy (PSP).^8^ The *C9ORF72* expansion instead may present not only with FTD but also with amyotrophic lateral sclerosis (ALS), or both, and is often associated with late onset psychosis.^9^

In FTD and ALS the lncRNAs nuclear paraspeckle assembly transcript 2 (NEAT2) and Metastasis Associated Lung Adenocarcinoma Transcript 1 (MALAT1) co-localize at nuclear paraspeckles with TDP-43 and FUS proteins.^10^ Moreover, the binding to TDP-43 is markedly higher in brains from demented patients. ^10^ The expression of 84 lncRNAs was analyzed in serum samples from genetic and sporadic FTD. Despite the statistical threshold was not reached due to limited sample size, the results showed a generalized deregulation of lncRNA expression levels in both genetic and sporadic FTD as compared with non-demented controls. In detail, a trend toward downregulation was observed in *GRN* and *C9ORF72* patients, whereas a trend toward upregulation was observed in *MAPT* mutation carriers. Notably, a few lncRNAs, including hepatocellular carcinoma upregulated EZH2-associated (HEIH), Eosinophil Granule Ontogeny Transcript (EGOT), and NEAT1, were downregulated in all groups.^11^ The origin of circulating lncRNAs is not known and no studies on lncRNA in circulating cells are available so far.

Here, we show results of profiling of lncRNAs in peripheral blood mononuclear cells (PBMC) from the very well characterized genetic GENFI cohort. To our knowledge, a large study of these molecules has not been carried out yet in genetic FTD, which represent the best model of the disease as the pathology can be predicted in life.

## MATERIALS AND METHODS

### Population

All demographic and clinical data, as well as samples included in the study, were collected within the Genetic frontotemporal dementia initiative (GENFI), a natural history study of genetic FTD involving several research centers across Europe and Canada (http://www.genfi.org.uk).^12^ The GENFI study was performed in accordance with the Declaration of Helsinki, reviewed and approved by all countries’ respective Ethics Committees and all participants signed an informed consent to take part in the research. This research study was performed in Italy, Ethics Committee Milano Area 2, parere 882_2022 del 13-9-22.

Variables included were: age at sampling, age at onset, Gender, mutation group (symptomatic mutation carriers, SMC; presymptomatic mutation carriers, PMC; non-carrier family members considered as controls, NC), mutated gene (*MAPT*, *GRN*, *C9ORF72*). One hundred and seventy-one PAX gene samples were collected, including 68 SMC (22 *MAPT*, 22 *GRN*, and 24 *C9ORF72*), 63 PMC (20 *MAPT*, 22 *GRN*, and 21 *C9ORF72*) and 40 NC. Carriers of other rare FTD causing mutations were not included. Demographics of the population are shown in Table 1.

**Table 1:** Demographic and clinical characteristics of the study population

	Non carriers (NC)	Presymptomatic mutation carriers (PMC)	Symptomatic mutation carriers (SMC)
Subjects (*n*)	40	63	68
Age, mean years (SD)	53 (15)	42 (11)	65 (8)
Age at onset, mean years (SD)	–	–	61 (8)
Gender distribution, females:males	27 : 13	36 : 27	28 : 40
Mutated gene *n* (%)
*GRN*		22 (35)	22 (32)
*C9ORF72*		21 (33)	24 (36)
MAPT		20 (32)	22 (32)
Age at onset, mean years (SD)
*GRN*			64 (7)
*C9ORF72*			59 (9)
*MAPT*			54 (6)
NfLpg/ml (SEM)	8.53 (0.18)	16.21 (2.83)	56.05 (3.81)^*^
GFAP pg/ml (SEM)	103.15 (10.21)	107.42 (6.12)	175.44 (9.32)^**^

^*^p = 0.002; ^**^p < 0.001.

### Sample processing

PAX gene tubes were stored and frozen according to the manufacturer. RNA was extracted using the PAX Gene blood miRNA kit that enable the extraction of total RNA including small ncRNA, according to the protocol of the manufacturer (Qiagen).

The quality and quantity of the extracted RNA were assessed using a Bioanalyzer 2100. The RNA Integrity Number (RIN) was determined, with values ranging from 7.9 to 8.4 across the samples, indicating that the RNA is of sufficient quality for further analysis (Supplementary Figure 1). Extracted RNA was stored at –80°C until use.

### Retrotranscription and real-time PCR

Total RNA was retrotranscribed with the SuperScript VILO cDNA Synthesis Kit. The OpenArray Custom panel, comprising a total of 56 lncRNA genes (53 target genes and three housekeeping genes: *B2M*, *ACTB* and *GAPDH*, Supplementary Table 1) was used following the manufacturer’s instructions (Thermofisher, 2012). The reproducibility between real-time PCR experiments was assessed by including the same cDNA sample in each run.

### Plasma neurofilament light chain and glial fibrillary acidic protein

Plasma neurofilament light chain (NfL) and glial fibrillary acidic protein (GFAP) levels were measured on the SIMOA HD-1 Analyzer as previously described.^13^

### Statistical analysis

Data were analyzed using the statistical spreadsheets Jamovi v 2.5.6.0 (https://www.jamovi.org). LncRNA expression levels (expressed as replicate 2^–ΔCt^ values for each gene) and gender were considered continuous variables and expressed as mean±standard deviation (SD). Conversely, gender, biological group (no mutations, *MAPT*, *C9ORF72*, and *GRN*, respectively) and genetic status (NC, PMC, and SMC, respectively) were all considered categorical variables. Significant statistical threshold was set at 0.05. Shapiro–Wilk’s test of normality was performed for continuous variables, and intergroup comparisons were carried out using ANCOVA test (gender and age as covariates). Post hoc analyses for multiple comparisons were performed with Tukey’s correction. The Pearson test was applied for correlations between deregulated lncRNAs and NFL and GFAP protein levels.

## RESULTS

Thirty-eight lncRNAs out of 53 were detectable. Among those, the relative expression of two lncRNAs, NEAT 1 and NORAD, were upregulated in *C9ORF72* biological group.

**Fig. 1:**
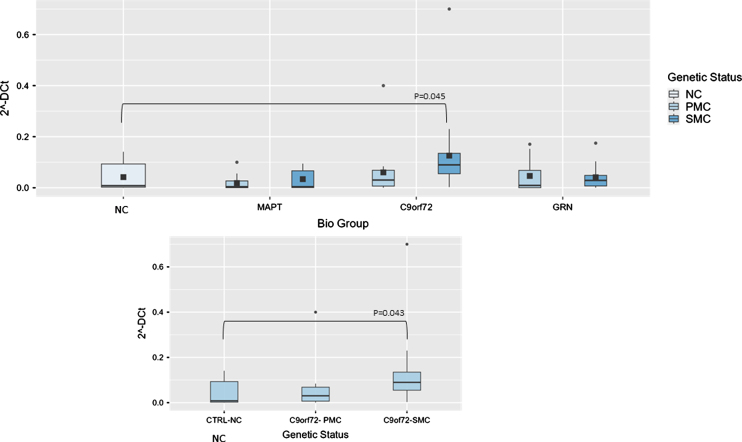
Box plots of NEAT1 profiling in PBMC from SMC and PMC carrying mutations in *C9ORF72, GRN, MAPT* as compared with NC. Data are expressed as 2^-ΔCt^ fold regulation.

In particular, the ANCOVA analysis of NEAT1 showed that the overall model yielded a significant result (*p* = 0.003), indicating that these variables collectively explain a significant portion of the variance in 2^–ΔCt^. Biological group showed a significant effect (F(3,96) = 5.35, *p* = 0.003, ἠ^2^*p* = 0.143), suggesting differences in 2^–ΔCt^ levels among the groups. Specifically, the *C9ORF72* group differed significantly from others in post hoc comparisons (0.098±0.01 versus 0.04±0.05-fold regulation, t(96) = –2.66, *p* = 0.043) compared to NC. As expected, age also had a significant influence (*p* = 0.046), indicating that older age was associated with altered 2^-Δ^ Ct levels, but the interaction between biological group and age was not statistically significant (*p* = 0.256). Stratifying results according to genetic status, the comparison between *C9ORF72* SMC and NC showed a statistically increased of NEAT1 2^–ΔCt^ values (0.12±0.01 versus 0.04±0.01-fold regulation versus NC, t(43) = –2.49, *p* = 0.043, Fig. 1).

**Fig. 2:**
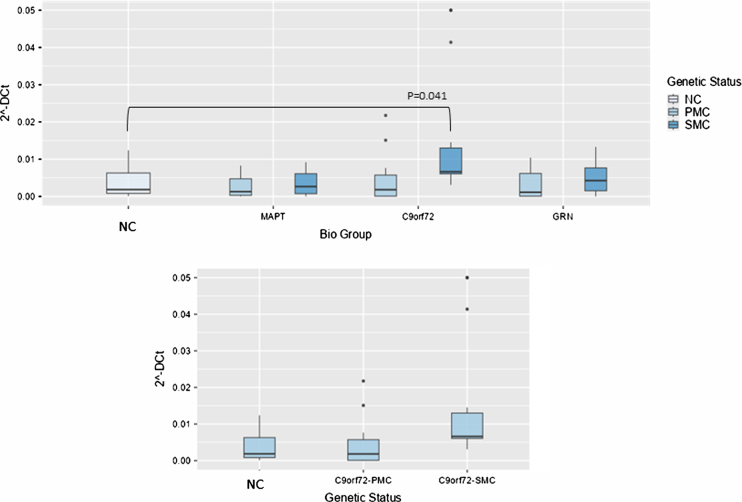
Box plots of NORAD profiling in PBMC from SMC and PMC carrying mutations in *C9ORF72, GRN, MAPT* as compared with NC. Data are expressed as 2^-ΔCt^ fold regulation.

**Fig. 3:**
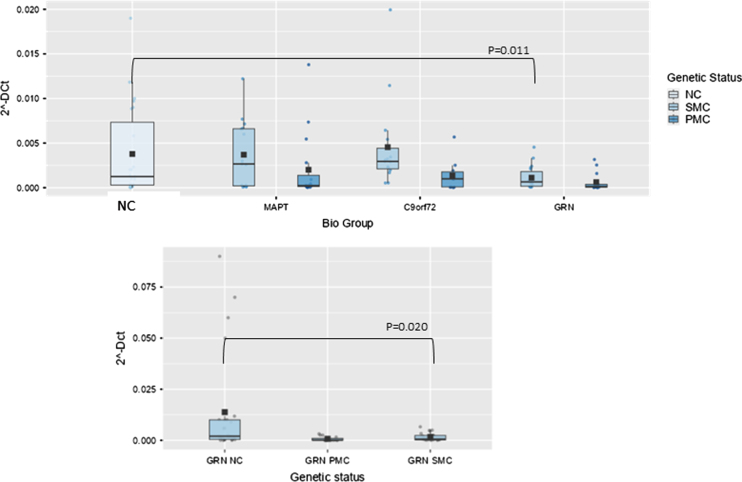
Box plots of GAS5 profiling in PBMC from SMC and PMC carrying mutations in *C9ORF72, GRN, MAPT* as compared with NC. Data are expressed as 2^-ΔCt^ fold regulation.

Concerning NORAD analysis, the overall model was significant (*P* < 0.001), indicating substantial variability explained by these variables. Biological group significantly impacted 2^–ΔCt^ levels (F(3,99) = 5.61, *p* = 0.001, ἠ^2^*p*=0.145), highlighting variability across NC, *MAPT*, *C9ORF72*, and *GRN* groups. Post hoc tests revealed that NORAD was significantly more abundant in *C9ORF72* biological group as compared with NC (0.014±0.01 versus 0.0038±0.00391-fold regulation, t(99) = –2.49, *p* = 0.041), but stratifying according to genetic status, the comparison between NC and *C9ORF72* SMC showed a trend towards significance (*p* = 0.065), suggesting potentially meaningful differences (Fig. 3).

In *GRN* biological group, one lncRNA, namely Growth Arrest Specific (GAS)5 was significantly lower than NC. The overall model was significant, indicating that the predictors collectively explain a significant portion of the variance in the dependent variable (*p* < 0.001). Age had a significant effect (*p* < 0.001), as did the biological group (F(3,105) = 3.65, *p* = 0.015, ἠ^2^*p*=0.095). However, nor did the interaction between biological group and age (*p* = 0.142). Post hoc comparisons using the Tukey test revealed that the *GRN* group significantly differed from NC (9.20×10^–4^ ^±^ 0.0011 versus 0.037×10^–2^ ^±^ 0.1×10^–3,^ fold regulation, t(105) = 3.14, *p* = 0.025). In *GRN* biological group GAS5 was significantly lower, also stratifying results according to genetic status and after post hoc analysis (SMC versus NC; 0.00164±0.0019 versus 0.013±0.024, fold regulation, t(55) = 2.78, *p* = 0.020, Fig. 3).

Conversely, no significant lncRNA deregulations were observed in *MAPT* carriers, both SMC and PMC, versus NC.

No correlations between disease duration in symptomatic individuals and lncRNA relative expression was found (*p* > 0.05).

Considering NfL and GFAP levels, as expected, significant increases were observed between SMC and NC: NfL mean levels±SEM were 56.05±3,81 versus 8.53±0.18 pg/ml, *p* = 0.002 and GFAP were 175.44±9.32 versus 103.15±10.21 pg/ml, *p* < 0.0001, respectively (Table 1). These differences in mean values remained significant even when stratifying symptomatic patients by the mutated gene (*p* < 0.001).

Considering instead the levels of NfL or GFAP and the lncRNAs, no significant correlations emerged (*p* > 0.05).

## DISCUSSION

Herein, we showed that the relative expression of NORAD and NEAT1 lncRNAs was higher in *C9ORF72* SMC, whereas relative levels of GAS5 were less abundant in *GRN* SMC. No significant differences were instead observed in PMC, although this group was very heterogenous as regards the age at sampling, and many subjects were still far from the mean age at disease onset in their families. Therefore, we cannot rule out whether the change observed in SMC occur in proximity of symptom manifestation.

LncRNA NORAD modulates the genome stability. It is expressed in cells after DNA damage and, when deleted, it expresses chromosomal instability and aneuploidy.^14^ Notably, *C9ORF72* expansions are known to cause R-loops, in turn increasing genomic instability and DNA damage, and generate dipeptide repeat proteins, that lead to DNA damage and impairment of the DNA damage response.^15^ Therefore, DNA damage may play a role in the pathogenesis of the disease in carriers of the hexanucleotide expansion. Here, we considered only patients with FTD, therefore we cannot rule out whether the same modifications are present also in carriers developing ALS. It is interesting to note that dendritic cells isolated from C9ORF72–/– mice showed a marked early activation of the type I interferon response, and C9ORF72–/– myeloid cells were selectively hyperresponsive to activators of the stimulator of interferon genes (STING) protein, key regulator of the innate immune response.^16^

As regards lncRNA NEAT1, in samples from patients with AD, it was shown that NEAT1 expression is upregulated in different brain regions related to the disease.^17^ Nevertheless, there are still controversial data on the role of this lncRNA (damaging versus protective) in AD and other neurodegenerative diseases. (see^18^ for review)

LncRNA GAS5 has been shown to be downregulated in brain samples from patients with AD compared to age-matched healthy controls.^19^ In line with this observation, a previous human transcriptomic analysis and microarray data of six brain regions from AD patients also showed that GAS5 is downregulated in AD.^20^

In a previous study, circulating levels of lncRNA were evaluated but, despite trends toward increased or decreased levels were observed, no significant data were obtained.^11^ It has to be considered however that the source of circulating lncRNA cannot be determined and that there are no study comparing PBMC and circulating lncRNA levels in the same individuals.

A strength of the study is represented by NC from the same families, reducing the genetic background variability. A weakness of the study is instead that we did not compare the same subjects from the asymptomatic to the symptomatic phase. As regards the longitudinal analysis of PMC, the follow up of participants in GENFI is ongoing and these findings could be confirmed longitudinally in the next future.

Lastly, it has to be acknowledged that, despite the extracted RNA being of good quality for subsequent expression analysis, several long non-coding RNAs were not detected. The lack of detection of long non-coding RNAs (lncRNAs) in blood can be attributed to several factors.

Firstly, the presence of ribonuclease enzymes in blood can lead to the rapid degradation of RNA molecules, including lncRNAs, making them difficult to detect.^21^

Additionally, many lncRNAs are expressed at intrinsically low levels, which may render them below the detection limit of standard real time PCR.^22^ Another significant factor is the tissue-specific expression of many lncRNAs: if a lncRNA is predominantly expressed in specific tissues such as the brain or liver, it is unlikely to be detected in the blood.^23^ RNA stability is another crucial element to consider, as some lncRNAs may be inherently unstable in blood, despite protective mechanisms such as encapsulation in exosomes or association with proteins, leading to their rapid degradation.^24^ These combined factors can explain the difficulty in detecting lncRNAs in blood. Therefore, we cannot rule out whether these lncRNAs play a role in neurodegeneration, and the present results need to be validated in an independent cohort of patients, possibly also including sporadic cases.

### Conclusions

Relative expression of NORAD and NEAT1 lncRNAs is more abundant in PBMC from *C9ORF72* SMC, whereas relative transcription of GAS5 is less abundant in PBMC from *GRN* SMC, whereas no significant changes have been observed in PMC. This study emphasizes the importance of lncRNAs in FTD and their potential for guiding genetic stratification and developing biomarkers. Further validation and mechanistic investigations are essential to facilitate clinical application.

## AUTHOR CONTRIBUTIONS

Serpente Maria (Data curation; Methodology; Validation; Writing – review & editing); Chiara Fenoglio (Conceptualization; Data curation; Formal analysis; Writing – review & editing); Marina Arcaro (Investigation; Methodology); Tiziana Carandini (Resources); Luca Sacchi (Resources); Manuela Pintus (Resources); Emanuela Rotondo (Project administration; Resources); Vittoria Borracci (Project administration; Resources); Laura Ghezzi (Resources); Arabella Bouzigues (Project administration; Resources); Lucy Russel (Project administration; Resources); Phoebe Foster (Resources); Eve Ferry-Bolder (Project administration; Resources); John van Swieten (Resources); Lize Jiskoot (Resources); Harro Seelaar (Resources); Raquel Sanchez-Valle (Resources); Robert laforce (Resources); Caroline Graff (Resources); Rik Vandenberghe (Resources); Alexandre de Mendonca (Resources); Pietro Tiraboschi (Resources); Isabel Santana (Resources); Alexander Gerhard (Resources); Johannes Levin (Resources); Sandro Sorbi (Resources); Markus Otto (Resources); Florence Pasquier (Resources); Simon Ducharme (Resources); Chris Butler (Resources); Isabelle Le Ber (Resources); Elizabeth Finger (Resources); Maria Carmela Tartaglia (Resources); Mario Masellis (Resources); James Rowe (Resources); Matthis Synofzik (Resources); Fermin Moreno (Resources); Barbara Borroni (Resources); Jonathan Rohrer (Project administration; Supervision); Andrea Arighi (Supervision; Writing – review & editing); Daniela Galimberti (Conceptualization; Project administration; Supervision; Writing – original draft; Writing – review & editing).

## Supplementary Material

Supplementary Material

## Data Availability

The datasets used and analyzed during the current study are available from the corresponding author on reasonable request.

## References

[1] Mattick JS , Amaral PP , Carninci P , et al. Long non-coding RNAs: Definitions, functions, challenges and recommendations. Nat Rev Mol Cell Biol 2023; 24: 430–447. 36596869 10.1038/s41580-022-00566-8 PMC10213152

[2] Iyer MK , Niknafs YS , Malik R , et al. The landscape of long noncoding RNAs in the human transcriptome. Nat Genet 2015; 47: 199–208. 25599403 10.1038/ng.3192 PMC4417758

[3] Faghihi MA , Modarresi F , Khalil AM , et al. Expression of a noncoding RNA is elevated in Alzheimer’s disease and drives rapid feed-forward regulation of beta-secretase. Nat Med 2008; 14: 723–730. 18587408 10.1038/nm1784 PMC2826895

[4] Li H , Zheng L , Jiang A , et al. Identification of the biological affection of long noncoding RNA BC200 in Alzheimer’s disease. Neuroreport 2018; 29: 1061–1067. 29979260 10.1097/WNR.0000000000001057

[5] Teixeira LCR , Mamede I , Luizon MR , et al. Role of long non-coding RNAs in the pathophysiology of Alzheimer’s disease and other dementias. Mol Biol Rep 2024; 51: 270. 38302810 10.1007/s11033-023-09178-7

[6] Pottier C , Ravenscroft TA , Sanchez-Contreras M , et al. Genetics of FTLD: Overview and what else we can expect from genetic studies. J Neurochem 2016; 138(Suppl 1), 32–53. 27009575 10.1111/jnc.13622

[7] Swift IJ , Rademakers R , Finch N , et al. A systematic review of progranulin concentrations in biofluids in over 7,000 people-assessing the pathogenicity of GRN mutations and other influencing factors. Alzheimers Res Ther 2024; 16: 66. 38539243 10.1186/s13195-024-01420-z PMC10976725

[8] Rademakers R , Neumann M , Mackenzie IR . Advances in understanding the molecular basis of frontotemporal dementia. Nat Rev Neurol 2012; 8: 423–434. 22732773 10.1038/nrneurol.2012.117 PMC3629543

[9] Galimberti D , Fenoglio C , Serpente M , et al. Autosomal dominant frontotemporal lobar degeneration due to the C9ORF72 hexanucleotide repeat expansion: Late-onset psychotic clinical presentation. Biol Psychiatry 2013; 74: 384–391. 23473366 10.1016/j.biopsych.2013.01.031

[10] Riva P , Ratti A , Venturin M . The long non-coding RNAs in neurodegenerative diseases: Novel mechanisms of pathogenesis. Curr Alzheimer Res 2016; 13: 1219–1231. 27338628 10.2174/1567205013666160622112234

[11] Fenoglio C , Serpente M , Visconte C , et al. Circulating non-coding RNA levels are altered in autosomal dominant frontotemporal dementia. Int J Mol Sci 2022; 23: 14723. 36499048 10.3390/ijms232314723 PMC9737170

[12] Rohrer JD , Nicholas JM , Cash DM , et al. Presymptomatic cognitive and neuroanatomical changes in genetic frontotemporal dementia in the Genetic Frontotemporal dementia Initiative (GENFI) study: A cross-sectional analysis. Lancet Neurol 2015; 14: 253–262. 25662776 10.1016/S1474-4422(14)70324-2 PMC6742501

[13] Heller C , Foiani MS , Moore K , et al. Plasma glial fibrillary acidic protein is raised in progranulin-associated frontotemporal dementia. J Neurol Neurosurg Psychiatry 2020; 91: 263–270. 31937580 10.1136/jnnp-2019-321954

[14] Huarte M . The emerging role of lncRNAs in cancer. Nat Med 2015; 21: 1253–1261. 26540387 10.1038/nm.3981

[15] Kok JR , Palminha NM , Dos Santos Souza C , et al. DNA damage as a mechanism of neurodegeneration in ALS and a contributor to astrocyte toxicity. Cell Mol Life Sci 2021; 78: 5707–5729. 34173837 10.1007/s00018-021-03872-0 PMC8316199

[16] McCauley ME , O’Rourke JG , Yáñez A , et al. C9orf72 in myeloid cells suppresses STING-induced inflammation. Nature 2020; 585: 96–101. 32814898 10.1038/s41586-020-2625-x PMC7484469

[17] Wu J , Chen L , Zheng C , et al. Co-expression network analysis revealing the potential regulatory roles of lncRNAs in Alzheimer’s disease. Interdiscip Sci 2019; 11: 645–654. 30715720 10.1007/s12539-019-00319-w

[18] Li K , Wang Z . lncRNA NEAT Key player in neurodegenerative diseases. Ageing Res Rev 2023; 86: 101878. 36738893 10.1016/j.arr.2023.101878

[19] Patel RS , Lui A , Hudson C , et al. Small molecule targeting long noncoding RNA GAS5 administered intranasally improves neuronal insulin signaling and decreases neuroinflammation in an aged mouse model. Sci Rep 2023; 13: 317. 36609440 10.1038/s41598-022-27126-6 PMC9822944

[20] Liang WS , Dunckley T , Beach TG , et al. Gene expression profiles in anatomically and functionally distinct regions of the normal aged human brain. Physiol Genomics 2007; 28: 311–322. 17077275 10.1152/physiolgenomics.00208.2006 PMC2259385

[21] Statello L , Guo C-J , Chen L-L , et al. Gene regulation by long non-coding RNAs and its biological functions. Nat Rev Mol Cell Biol 2021; 22: 96–118. 33353982 10.1038/s41580-020-00315-9 PMC7754182

[22] Dehling E , Volkmann G , Matern JCJ , et al. Mapping of the communication-mediating interface in nonribosomal peptide synthetases using a genetically encoded photocrosslinker supports an upside-down helix-hand motif. J Mol Biol 2016; 428: 4345–4360. 27647046 10.1016/j.jmb.2016.09.007

[23] Baldock RA , Day M , Wilkinson OJ , et al. ATM localization and heterochromatin repair depend on direct interaction of the 53BP1-BRCT2 domain with *γ*H2AX. Cell Rep 2015; 13: 2081–2089. 26628370 10.1016/j.celrep.2015.10.074 PMC4688034

[24] Managadze D , Rogozin IB , Chernikova D , et al. Negative correlation between expression level and evolutionary rate of long intergenic noncoding RNAs. Genome Biol Evol 2011; 3: 1390–1404. 22071789 10.1093/gbe/evr116 PMC3242500

