# Supplementary material for: Long Non-Coding RNA Profile in Genetic Symptomatic and Presymptomatic Frontotemporal Dementia: A GENFI Study
Source: J Alzheimers Dis. 2024 Aug 20;100(Suppl 1):S187–96. doi: 10.3233/JAD-240557 (PMC11380264; doi:10.3233/JAD-240557)
Supplement: Supplementary Material [file jad-100-jad240557-s001.pdf]

Supplementary Material

Long Non-Coding RNA Profile in Genetic Symptomatic and Presymptomatic Frontotemporal Dementia: A GENFI Study

Supplementary Table 1. Gene List

**Species**	
Homo sapiens	
**lncRNA Symbol**	**Assay ID**
**BCYRN1**	Hs04404041_m1
**KCNQ1DN**	Hs00218884_m1
**BACE1-AS**	Hs04232267_s1
**HLA-AS1**	Hs03678084_m1
**HAR1A**	Hs05038333_s1
**HAR1B**	Hs03299152_m1
**LRP1-AS**	Hs04937844_m1
**MIAT**	Hs03300285_g1
**SOX2-OT**	Hs00415716_m1
**IFNG-AS1**	Hs04408310_m1
**DLX6-AS1**	Hs03680408_m1
**XIST**	Hs01079824_m1
**H19**	Hs00262142_g1
**HOTAIR**	Hs03296631_m1
**MALAT1**	Hs00273907_s1
**MAPT-AS1**	Hs03681077_s1
**NEAT1**	Hs03453535_s1
**PCA3**	Hs01371939_g1
**PINK1-AS**	Hs04404903_m1
**SNHG1**	Hs00411543_m1
**BDNF-AS**	Hs03298540_m1
**DISC1**	Hs00962132_m1
**DISC2**	Hs03297678_s1
**LINC00271**	Hs03666572_m1
**Meg3**	Hs00292028_m1
**ATG10-AS**	Hs04977857_s1
**SOX21AS1**	Hs01382396_g1

**lncRNA Symbol**	**Assay ID**
**TUG1**	Hs00215501_m1
**ANRIL**	Hs01390879_m1
**LINC00173**	Hs00858478_m1
**A2MAS1**	Hs01394095_g1
**OIP5-AS1**	Hs05010043_s1
**LINC00960**	Hs01572486_m1
**TCF7L2**	Hs01009044_m1
**ACER2**	Hs03866108_s1
**LINC00507**	Hs01387470_m1
**GAS5**	Hs03464472_m1
**UCHL1**	Hs00985157_m1
**CDR1AS**	Hs05016408_s1
**OIP5-AS**	Hs03677189_g1
**NORAD**	Hs05023184_s1
**B2M**	Hs00187842_m1
**GAPDH**	Hs02786624_g1
**ACTB**	Hs01060665_g1
**GDNF-AS1**	Hs04939841_g1
**CCAT2**	Hs04403001_s1
**FAS-AS1**	Hs04233476_s1
**LINC01262**	Hs05044879_g1
**NRON**	Hs04274937_s1
**lincRNA-COX2**	APMF4VA
**MINCR**	Hs00935703_s1
**51A**	APNKYE7
**UCA1**	Hs01909129_s1
**WFDC21P**	Hs01371942_m1
**PANDAR**	Hs01659750_sH
**THRIL**	Hs04404410_s1

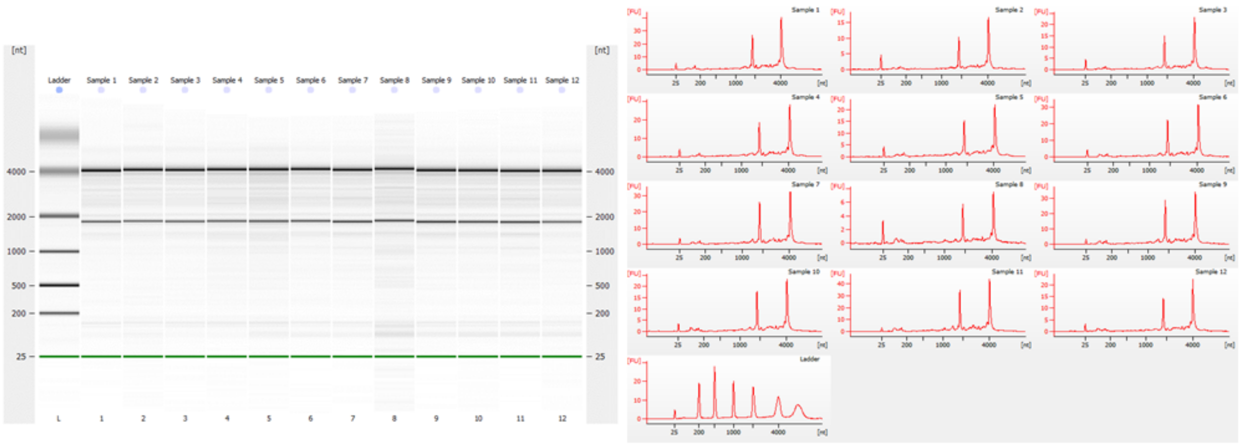

Supplementary Figure 1. An example of electropherogram images from a Bioanalyzer 2100 run, showing RNA extracted from a group of subjects included in the study. Each peak corresponds to a different size fragment, and the height of the peak indicates the abundance of that fragment.
